# Sodium-Glucose Linked Transporter-2 Inhibitors in Chronic Kidney Disease

**DOI:** 10.1155/2015/317507

**Published:** 2015-02-15

**Authors:** L. Zanoli, A. Granata, P. Lentini, S. Rastelli, P. Fatuzzo, F. Rapisarda, P. Castellino

**Affiliations:** ^1^Department of Internal Medicine, University of Catania, 95100 Catania, Italy; ^2^Department of Nephrology, Presidio Ospedaliero Agrigento, 92100 Agrigento, Italy; ^3^Department of Nephrology, San Bassiano Hospital, 36061 Bassano del Grappa, Italy

## Abstract

SGLT2 inhibitors are new antihyperglycaemic agents whose ability to lower glucose is directly proportional to GFR. Therefore, in chronic kidney disease (CKD) the blood glucose lowering effect is reduced. Unlike many current therapies, the mechanism of action of SGLT2 inhibitors is independent of insulin action or beta-cell function. In addition, the mechanism of action of SGLT2 inhibitors is complementary and not alternative to other antidiabetic agents. SGLT2 inhibitors could be potentially effective in attenuating renal hyperfiltration and, consequently, the progression of CKD. Moreover, the reductions in intraglomerular pressure, systemic blood pressure, and uric acid levels induced by SGLT inhibition may potentially be of benefit in CKD subjects without diabetes. However, at present, only few clinical studies were designed to evaluate the effects of SGLT2 inhibitors in CKD. Consequently, safety and potential efficacy beyond blood glucose lowering should be better clarified in CKD. In this paper we provide an updated review of the use of SGLT2 inhibitors in clinical practice, with particular attention on subjects with CKD.

## 1. Introduction

Diabetes is one of the major health problems worldwide, with a prevalence that is expected to reach more than 550 million patients by 2030 [1], and it is one of the leading causes of renal disease resulting in a very high prevalence among dialysis patients [2]. It is estimated that approximately one-third of patients with type 2 diabetes mellitus (T2DM) have some degree of renal impairment [3] and that chronic kidney disease (CKD) is detectable in an important part of diabetic patients [4]. CKD is characterized by a permanent loss of nephron units. The loss of renal mass induces a compensatory condition of glomerular hyperfiltration and tubular hypertrophy which in turn may lead to glomerular sclerosis with the attendant progression to end stage renal disease.

Results from randomized controlled trials have demonstrated that the risk of microvascular complications, including retinopathy, neuropathy, and nephropathy, can be reduced by intensive glycaemic control in patients with type 2 diabetes mellitus [5, 6]. However, only about half of diabetic patients achieve the recommended glycaemic goals. Moreover, because in most patients with T2DM beta-cell function continues to decline as their disease progresses, over time, higher doses and additional diabetes medications are necessary to achieve and maintain glycaemic goals. Accordingly, medications that rely on insulin secretion are characterized by a high rate of secondary failure and can be used only in the initial phases of diabetes, and most patients will ultimately rely on insulin for glycaemic control. New therapies with complementary mechanisms of action that are independent of insulin secretion or action and have acceptable safety profiles, such as sodium-glucose linked transporter-2 (SGLT2) inhibitors, may provide additional therapeutic options to achieve glycaemic control and renoprotection.

## 2. Oral Glucose-Lowering Agents in Chronic Kidney Disease

In CKD subjects with glomerular filtration rate 15–60 mL/min/1.73 m^2^ (stages III-IV KDOQI), the use of oral antidiabetic agents should be carefully monitored because it is frequently necessary as a dose adjustment, or because they are contraindicated for safety reasons in particular. Metformin, which represents a corner stone in the treatment of patients with type II diabetes, should be used with caution in CKD patients due to the risk of accumulation and lactic acidosis. When GFR declines below 30 mL/min, it should be discontinued. A similar caution is advised with the use of insulin secretagogues in CKD patients because the dipeptidyl peptidase-4 (DPP-4) inhibitors saxagliptin, sitagliptin, and vildagliptin (but not linagliptin) are predominantly excreted by kidneys; therefore, dose reduction is necessary in patients with CKD. The use of insulin secretagogues is associated in CKD patients with a higher rate of hypoglycaemic episodes and sudden death. Finally, despite the lowering effect of pioglitazone on microalbuminuria, all-cause mortality, myocardial infarction, and stroke in CKD patients, as well as the low risk of hypoglycaemia, this drug should be used with caution in CKD stages 4-5 for the increased risk of water and sodium retention and heart failure.

## 3. SGLT2 Inhibitors

The kidney plays an important role in glucose homeostasis, mostly by the reabsorption of filtered glucose. In the kidney, filtered glucose is actively reabsorbed by specific transporters located on the apical (brush-border) membrane of proximal tubular cells. Two different types of Na^+^/glucose transporters, SGLT1 and SGLT2, are expressed in the kidney and mediate the renal handling of glucose. SGLT2 is located in S1 segment of the proximal tubule and it is responsible for the majority of the glucose reabsorption (80–90%) [11]. It is a low-affinity/high capacity system, whereas SGLT1 absorbs the remnant 10–20% of the filtered glucose from the lumen of S3 segment as a high-affinity/low-capacity system. These findings are supported by observations that glucose reabsorption is reduced by approximately 60% in SGLT2-null mice, compared with normal mice [12], and that subjects with familial renal glycosuria, who have mutations in the gene coding for SGLT2, excrete significant amounts of glucose (up to ≥10 g/1.73 m^2^/day) in the absence of hyperglycaemia and with no evidence of generalized proximal tubule dysfunction [13].

In nondiabetic subjects all the filtered glucose is reabsorbed in the proximal tubule and virtually no glucose is present in the urine. However, the maximal renal glucose reabsorptive capacity (tubular max for glucose, T_mG_) varies among individuals and averages 375 mg/min [12, 14, 15] which with a normal GFR of 120 mL/min corresponds to a plasma glucose concentration of approximately 180 mg/dL. If the filtered glucose load exceeds the T_mG_, all glucose excess of the T_mG_ is excreted. This relationship explains why diabetic subjects usually develop glycosuria only when their plasma glucose exceeds 180–200 mg/dL (10–12 mmol/L). Glucose reabsorption and excretion curves display a nonlinear shift (splay) as T_mG_ is approached. The plasma glucose concentration at which glucose begins to appear in the urine is reported as threshold and corresponds to the splay. In diabetic subjects, both SGLT2 expression and T_mG_ (>400 mg/min) are increased. Therefore, in the presence of elevated glucose levels, the kidneys continue to reabsorb a large proportion of the filtered glucose and this contributes to the maintenance of hyperglycaemia. In a porcine model, 48 h after the administration of 450 mg/dL glucose to the apical compartment of porcine proximal tubular cells, intracellular glucose increased over threefold and this was associated with an intense stimulation of fibronectin secretion into the basolateral supernatant [16]. These findings suggest that elevations of luminal and interstitial glucose concentration contribute to proximal tubular extracellular matrix synthesis and, consequently, may explain the development of tubulointerstitial fibrosis in diabetic nephropathy. According to these findings, it has been reported that SGLT2 inhibition in human proximal tubular cells exposed to high glucose is associated with a reduction in inflammatory and fibrotic markers [17].

SGLT2 inhibitors are a new class of antihyperglycaemic drugs approved for the treatment of type 2 diabetes. Three highly selective SGLT2 inhibitors, dapagliflozin (FORXIGA 5–10 mg, Bristol-Myers Squibb, Princeton, NJ, and AstraZeneca, Wilmington, DE), canagliflozin (INVOKANA 100–300 mg, Janssen Pharmaceuticals, NJ, USA, and Janssen-Cilag International NV, Berchem, BE), and empagliflozin (JARDIANCE 10–25 mg, Boehringer Ingelheim Pharmaceuticals, Inc., CT, USA, Eli Lilly and Company, Indiana, USA), have been approved for patients use. These drugs offer a novel approach to the treatment of type 2 diabetes mellitus. Their effect is a reduction of hyperglycaemia secondary to the reduction of T_mG_ and the attendant increase of urinary glucose excretion. In addition to glucose lowering, SGLT2 inhibitors could be effective in attenuating renal hyperfiltration in subjects with type 1 diabetes [18]. It should be pointed out that, differently to many current therapies for diabetes, the mechanism of action of SGLT2 inhibitors is independent of insulin secretion or action and, therefore, it does not depend on beta-cell function. In addition, the mechanism of action of SGLT2 inhibitors is complementary and not alternative to the mechanisms of other antidiabetic agents. Thus, SGLT2 inhibitors may be suitable for use in a combination of approaches. Finally, because SGLT2 inhibitors increase glucose excretion and, therefore, calorie losses, they may cause a decrease in body weight. This may be advantageous since most therapies for diabetes such as insulin, insulin secretagogues, and thiazolidinediones are associated with an increase in food intake and weight gain. Even minor changes in weight are known to be associated with significant antihypertensive effects and may therefore contribute to the blood pressure lowering effect of SGLT2 inhibitors [19]. In meta-analyses of clinical trials comparing SGLT2 inhibitors with placebo or active treatments with metformin, sulfonylurea, DPP-4 inhibitor or insulin, and SGLT2 inhibitors reduced HbA1C by approximately 0.5–0.7% (mean difference versus active comparators −0.06%), making them relatively weak glucose-lowering agents, similar in potency to the DPP-4 inhibitors [20–22].

As far as side effects are concerned, SGLT2 inhibitors are well tolerated with a low rate of drug-induced hypoglycaemia. Other side effects are reported in the Efficacy and Safety Data of SGLT2 Inhibition. The final effect of the administration of SGLT2 inhibitors is a decline of single-nephron glomerular filtration rate (SNGFR). This decline may be determined by various and complex changes in tubuloglomerular feedback. It is well known that glomerular filtration and electrolyte reabsorption are finely coordinated within individual nephrons and minute-to-minute changes in the flow and composition of urine are sensed by the macula densa through the action of the Na^+^/K^+^/2Cl^−^ cotransporter, located between the distal loop of Henle and the early distal convoluted tubule, in close proximity to the afferent and efferent arterioles of the same nephron. In diabetes, increased proximal tubular glucose reabsorption is accompanied by augmented SGLT1/2-mediated glucose-Na^+^ cotransport, thus reducing the NaCl delivery at the macula densa. This is a potent signal for a compensatory increase of SNGFR via tubuloglomerular feedback which in turn will restore a normal distal sodium delivery. In diabetic rats, administration of a SGLT2 inhibitor led to an acute inhibition of proximal glucose-Na^+^ cotransport with a threefold increase in Na^+^ excretion and a 20% fall in GFR [23]. However, this effect is lost with chronic SGLT2 blockade, suggesting that an adaptive increase in Na^+^ reabsorption distal to the juxtaglomerular apparatus occurs in the long-term setting. The persistent 15% decline in GFR with chronic SGLT2 blockade [23] suggests that changes in tubuloglomerular feedback function are still present. Finally, similar to familial renal glycosuria, SGLT2 inhibition determines a reduction of effective circulating volume and an increase in circulating RAAS activity [24]. This observation could be of interest in subjects treated with RAAS inhibitors because it is well known that the use of RAAS blockers in combination with diuretics has synergistic antihypertensive and antiproteinuric effects. Similarly, RAAS blockade with SGLT2 inhibition, which induces natriuresis and volume contraction, has recently been shown in animals to have additive renoprotective effects in comparison to either drug alone [25].

## 4. SGLT2 Inhibitors in CKD

At present, only few studies have evaluated the effect of SGLT2 inhibition in CKD. For this reason, their use is not recommended in subjects with severe renal impairment or end-stage renal disease. This use in dialysis patients is obviously contraindicated. Moreover, the magnitude of glucose excretion and haemoglobin A1c reduction induced by SGLT2 inhibitors is dependent upon the filtered glucose load and is maximal in diabetic subjects with normal GFR and a high filtered glucose load and only modest in patients with renal impairment, in whom the filtered glucose load is reduced.

In addition, in 5/6 nephrectomized rats it has been reported that SGLT2 in the isolated renal apical (brush-border) membrane vesicles was markedly decreased in nephrectomized rats compared with controls [26] and that the mRNA expression levels of SGLT2, but not SGLT1, were markedly depressed, suggesting that loss of SGLT2 during CKD could reduce the single nephron maximal glucose reuptake and increase the risk of renal glycosuria.

Interestingly, it has been reported that empagliflozin fails to reduce urinary albumin excretion in diabetic mice with elevated blood glucose levels [27]. The authors suggested that lack of renoprotection could be the result of (a) incomplete inhibition of glucose reabsorption by empagliflozin or (b) the elevated glucose entry into the glomerular compartment (unmodified by SGLT2 inhibition) and consequent glomerular damage and CKD progression. In support of this hypothesis, it has been reported that SGLT2 inhibitors reduce diabetic nephropathy in type 2 diabetic rats with optimal glycaemic control [28].

To date, only three clinical studies examined the effects of SGLT2 inhibition on renoprotection and glycaemic control in CKD [7–10, 29] (Table 1). In study 1 [7], performed in subjects with CKD stage 3 (GFR 30–60 mL/min/1.73 m^2^), during the first 24 weeks of therapy GFR changed more in subjects treated with SGLT2 inhibitors than placebo (−2.8, −4.8, and −0.3 mL/min/1.73 m^2^ with 5 mg dapagliflozin, 10 mg dapagliflozin, and placebo, resp.) [7]. However, during the following 80 weeks of follow-up, a reverse pattern was observed in a small number of participants (+0.7, +1.3, and −2.1 mL/min/1.73 m^2^ in 4 placebo, 8 dapagliflozin 5 mg, and 10 dapagliflozin 10 mg group) [7]. This finding should be confirmed in a larger population. Accordingly, during the 24 weeks of follow-up, HbA1c was reduced by 0.38%, 0.41%, and 0.28% in dapagliflozin 5 mg, dapagliflozin 10 mg, and placebo group, respectively, with a further decline in all three groups at the end of follow-up. Interestingly, this study has not achieved its primary efficacy objective, a change in HbA1c from baseline to 24 weeks, reasonably due to an overestimation of the change in HbA1c in dapagliflozin group [8, 29]. In study 2 [8], when compared with baseline measurements, the urinary albumin/creatinine ratio fell by a median of 29.9%, 20.9%, and 7.5% in canagliflozin 100 mg, 300 mg, and placebo groups over the 6-month study period whereas HbA1c was reduced by 0.33%, 0.44%, and 0.03%, respectively [8]. The extension of follow-up to 52 weeks confirms that canagliflozin was well tolerated and improved glycaemic control, body weight, and blood pressure [9]. GFR decreased more in canagliflozin 100 and 300 mg group compared with placebo after 24 weeks of follow-up (−3.6, −3.9, and −1.8 mL/min/1.73 m^2^, resp.) and was almost unchanged after 52 weeks (−2.1, −4.0, and −1.6 mL/min/1.73 m^2^, resp.). Urinary albumin/creatinine ratio declined in both treatment groups and increased in controls (−16.4%, −28.0%, and 19.7%, resp.). Finally, in study 3 [10], HbA1c was reduced by 0.37% in empagliflozin 25 mg and did not change in placebo group. Taken together, these findings suggest that SGLT2 inhibitors may have a renoprotective effect with a decline in proteinuria and a long-term maintenance of GFR. However at present, it is not possible to determine if the abovementioned effect may have a long-term advantageous impact on the progression of diabetic nephropathy. Similarly, it is not possible to infer if the decline in proteinuria may be related to changes in intraglomerular hemodynamic and pressure, as demonstrated for ACEi and sartans, or if it may be ascribed to other action(s) of SGLT2 inhibitors on renal function. Interestingly, the pattern of an acute reduction followed by a stabilization in GFR reported in study 1 and study 2 in the SGLT2 inhibitors groups [7–10] is similar to those reported with angiotensin-converting enzyme inhibitors (ACEi) in subjects with CKD but with different underlying mechanisms for the changes in GFR.

## 5. Efficacy and Safety Data of SGLT2 Inhibition

Long-term efficacy and safety data on the use of SGLT2 inhibitors are still incomplete and their use in patients with type 2 diabetes should be carefully considered.

The most common side effects observed in patients treated with SGLT2 inhibitors are genital mycotic and urinary tract infections. It may be related to the prolonged and sustained glycosuria induced by SGLT2 inhibitors. These drugs reduce the tubular reabsorption of glucose and increase the osmotic diuresis, thus they can potentially cause dehydration, hypotension, and decline in renal function. Elderly patients with impaired renal function and those on treatment with diuretics are more susceptible to this risk.

Prior to dapagliflozin FDA approval, concerns were raised citing higher rates of breast and bladder cancer in subjects treated with the drug [30]. It should be pointed out that all but one of the patients with bladder cancer in the FDA analysis had evidence of haematuria before the use of dapagliflozin. These findings, coupled with the grade of the cancer at diagnosis, suggest that causation was questionable. Nevertheless, it is possible that high levels of glucose in the bladder induced by glycosuria may accelerate the rate of growth for preexisting cancers. These considerations led to the current statement of FDA that does not recommend dapagliflozin in patients with active bladder cancer. Postmarketing studies, focused on the evaluation of the risk of bladder cancer in patients treated with dapagliflozin, are clearly needed.

The use of SGLT2 inhibitors in CKD, increasing the risk of side effects, should be carefully considered. The glucose lowering benefit of empagliflozin decreased in CKD patients (GFR 30–90 mL/min/1.73 m^2^) as well as the risks of renal impairment, volume depletion adverse reactions, and urinary tract infection-related adverse reactions. The efficacy and safety of empagliflozin have not been established in patients with severe renal impairment (GFR < 30 mL/min/1.73 m^2^). The use of canagliflozin in patients with moderate CKD is less effective in improving glycemic control and is associated with a higher occurrence of adverse reactions related to reduced intravascular volume, renal-related adverse reactions, and decreases in GFR compared to patients with mild renal impairment or normal renal function. Finally, patients on treatment with canagliflozin should be evaluated for the higher risk for hyperkalemia. The use of dapagliflozin in patients with moderate CKD is not effective in improving glycaemic control and had more renal-related adverse reactions and more bone fractures and adverse reactions than placebo-treated patients. As reported by FDA [31], monitoring of bone health in clinical studies was in part prompted by the finding that clinically relevant doses of canagliflozin increased trabecular (cancellous) bone and decreased bone turnover in short- and long-term toxicology studies conducted on rats. Trabecular accretion was most prominent in younger rats with rapidly growing bones and was reversible upon cessation of drug exposure. Despite the increase in trabecular number and volume, bone mineralization and strength were not substantially altered at clinically relevant drug exposure. Measures of bone mineralization and strength at higher doses of canagliflozin were confounded by reductions in body weight from excessive diuresis and caloric loss.

Finally, intravascular volume depletion and the related potential adverse renal effects have been a concern with SGLT2 inhibitors. At this regard, an early and dose-dependent increase in serum creatinine levels was observed in subjects treated with SGLT2 inhibitors, in particular in those with renal impairment. This point has been extensively discussed in the SGLT2 Inhibitors in CKD.

## 6. Perspectives

In a T2DN rat model, chronic treatment with luseogliflozin normalizes blood glucose and HbA1c levels similarly to insulin-treated control animals [25]. Interestingly, luseogliflozin had no effect on blood pressure. Nevertheless, in combination therapy with lisinopril, an ACEi with a well-known renoprotective effect, it reduces glomerular injury, renal fibrosis, and tubular necrosis to a greater extent than administration of either drug alone [25]. RAAS inhibitors have a renoprotective effect which is mediated by a decline in efferent arteriolar tone and glomerular hypertension [32]. Recent evidences in diabetic SGLT2^−/−^ mice suggest that SGLT2 may affect afferent arteriolar tone in addition to a reduction of the proximal tubular SGLT1/2-mediated glucose reabsorption (resulting in lower extracellular matrix synthesis and tubulointerstitial fibrosis) and Na^+^ reabsorption (resulting in increased NaCl concentration at the macula densa and reduced GFR via tubuloglomerular feedback) [33]. If confirmed in clinical studies, these findings may add a valuable tool to achieve the renoprotection of subjects with CKD and diabetes. At present, the available therapeutic options to preserve the remaining nephrons in subjects with diabetic nephropathy are based on the use of RAAS blockers and on the achievement of a strict control of blood glucose level to reduce glomerular hyperfiltration and matrix synthesis. The modest effects on blood glucose suggest that use of SGLT2 inhibitors may have a renoprotective effect beyond the control of hyperglycaemia. Clinical studies focused on these issues are currently missing.

In conclusion, SGLT2 inhibitors are a new class of antidiabetic drugs which induce a moderate effect on blood glucose, especially in CKD. Further studies are needed to look whether SGLT2 inhibitors have renoprotective effects beyond the control of hyperglycaemia in subjects with CKD. They may represent a significant additional therapeutic tool in the clinical prevention and management of diabetic nephropathy.

## Figures and Tables

**Table 1:** Glycaemic control and renoprotection in studies of SGLT2 inhibition in subjects with moderate chronic kidney disease.

Study 1 [7]	Placebo	Dapagliflozin 5 mg	Dapagliflozin 10 mg
HbA1c^*^			
Baseline, %	8.5	8.3	8.2
Changes at week 24, %	−0.28	−0.38	−0.41
Changes at week 104, %	−0.67	−1.21	−0.75
GFR			
Baseline, mL/min/1.73 m^2^	45.6	44.2	43.9
Changes at week 24, mL/min/1.73 m^2^	−0.3	−2.4	−4.8
Changes at week 104, mL/min/1.73 m^2^	−2.4	−1.7	−3.5

Study 2 [8, 9]	Placebo	Canagliflozin 100 mg	Canagliflozin 300 mg

HbA1c			
Baseline, %	8.0	7.9	8.0
Changes at week 26, %	−0.03	−0.33	−0.44
Changes at week 52, %	−0.07	−0.19	−0.33
GFR			
Baseline, mL/min/1.73 m^2^	40.1	39.7	38.5
Changes at week 26, mL/min/1.73 m^2^	−1.8	−3.6	−3.9
Changes at week 52, mL/min/1.73 m^2^	−1.6	−2.1	−4.0
Urinary albumin/creatinine ratio			
Baseline, *μ*g/mg	31.3	23.7	30.1
Changes at week 26, %	−7.5	−29.9	−20.9
Changes at week 52, %	19.7	−16.4	−28.0

Study 3 [10]	Placebo	Empagliflozin 25 mg	

HbA1c			
Baseline, %	8.0	8.0	
Changes at week 24, %	0.05	−0.37	

^*^No statistically significant change in hemoglobin A1c (HbA1c).
